# Bibliographical Department

**Published:** 1880-08

**Authors:** 


### BIBLIOGRAPHICAL DEPARTMENT.

"Nothing extenuate, nor set down aught in malice."

"A case of Ovariotomy in which Phlegmasia Dolens followed the operation, Vaccination, by Walter F. Atlee, M. D., of Philadelphia."

From the author, "Sixty-third Annual Report of the State of the Asylum for the Relief of Persons Deprived of the use of their Reason. (Friends Asylum for the insane,)" Philadelphia.

"Hinderances to Respiration from Disease of the Nose. By D. H. Goodwillie, M. D., D. D. S., New York City."

"Sixth annual Report of the Baltimore Charity Eye and Ear Dispensary. Dr. Samuel Theobald, Attending surgeon."

"What constitutes a Discovery in Science. By George M. Beard, M. D., A. M., New York," and

A Reply to Criticisms on "The Problems of Insanity," Remarks on the Gosling case, delivered before the New York Medico-Legal Society, April 16th, 1880." By the same author.

"The Early Application of the Forceps in the First Stage of Natural Labor. By Isaac E. Taylor, M. D., New York, from the Gynecological Transactions for 1880. "We regard this monograph by Professor Taylor, as a highly important and valuable contribution to the department of Gynecology or Obstetrics, and would be much pleased to hear from him further upon the subject in an article for the pages of this journal, as early as he may have time and inclination to give his experience to the profession. Want of space forbids our speaking of the other pamphlets as they severally merit.

"The Twenty-fifth Annual Announcement of the Pennsylvania College of Dental Surgery, Philadelphia," indicates the school in a prosperous condition.

"Announcement: Boston Dental College, 1880-81,"

"Ship Origin of Yellow Fever. By R. B. S. Hargis, M. D."

"Second Annual Announcemrnt of St. Joseph College of Physicians and Surgeons."

"St. Joseph Medical and Surgical Reporter, a monthly journal. Price $2 per annum, No. 1, Vol. 1, has been received, and presents a neat and interesting appearance. We wish it success.

